# Evidence on respectful maternity care for adolescents: a systematic review protocol

**DOI:** 10.1186/s13643-021-01829-9

**Published:** 2021-10-15

**Authors:** Helen H. Habib, Jefferson Mwaisaka, Kwasi Torpey, Ernest Tei Maya, Augustine Ankomah

**Affiliations:** 1 Department of Population, Family and Reproductive Health, School of Public Health, College of Health Sciences, University of Ghana, Accra, Ghana; 2 Population Council Ghana, Accra, Ghana

**Keywords:** Respectful Maternity Care, Adolescent sexual reproductive health and rights, Intra-partum mistreatment

## Abstract

**Background:**

Intrapartum mistreatment of women is an ubiquitous public health and human rights challenge. The issue reportedly has severe maternal and neonatal outcomes including mortality, and generally leads to a decreased satisfaction with maternity care. Intrapartum mistreatment, despite being ubiquitous, indicates higher incidence amongst adolescent parturients who are simultaneously at a higher risk of maternal morbidity and mortality. Studies have suggested that Respectful Maternity Care interventions reduce intrapartum mistreatment and improve clinical outcomes for women and neonates in general. However, evidence on the effect of RMC on adolescents is unclear. Hence, the specific aim of this study is to synthesise the available evidence relating to the provision of RMC for adolescents during childbirth.

**Methods:**

The methodology of the proposed systematic review follows the procedural guideline depicted in the preferred reporting items for systematic review protocol. The review will include published studies and gray literature from January 1, 1990, to June 30, 2021. Electronic databases including MEDLINE, PubMed, ScienceDirect, Cochrane, CINAHL, PsycINFO, Scopus, Google Scholar and Web of Science will be searched to retrieve available studies using the appropriate search strings. Studies included in the review will be appraised for quality using tools tailored to each study design. If appropriate, we will conduct random effects meta-analysis of data to summarise the pooled estimates of respectful maternity care prevalence and outcomes. The selection of relevant studies, data extraction and quality assessment of individual studies will be carried out by two independent authors.

**Results:**

Summaries of the findings will be compiled and synthesised in a narrative summary. In addition to the narrative synthesis, where sufficient data are available, a random-effects meta-analysis will be conducted to obtain a pooled estimate value for respectful maternity care prevalence and outcomes.

**Discussion:**

Respectful Maternity Care for adolescents holds great promise for improved maternal and neonatal care. However, there is a gap in knowledge on the interventions that work and the extent of their effectiveness. Findings from this study will be beneficial in improving Adolescents Sexual and Reproductive Health and Rights and reducing maternal mortality, especially for adolescents.

**Systematic review registration:**

PROSPERO CRD42020183440

**Supplementary Information:**

The online version contains supplementary material available at 10.1186/s13643-021-01829-9.

## Background

Intrapartum mistreatment during facility-based deliveries is a severe, albeit ubiquitous predicament faced by parturient women worldwide [1, 2]. Mistreatment is classified as both a public health and human rights issue [3]. It infringes on the rights to health of women and is strongly linked to health outcomes like maternal and neonatal morbidity and mortality with some effects lasting chronically until further into the life course [4].

Research has focused on investigating efficacious strategies which successfully alleviates this challenge. Efforts to reduce maternal and neonatal mortality and morbidity have identified and recommended facility-based births and as a corollary, increasing the proportion of births attended by skilled birth attendants to enable early identification and immediate management of arising complications [5]. Although trends in facility-based births across sub-Saharan Africa have generally showed upward trajectory over the past decade, uptake is still less than universal and many women have reported reluctance to use facilities due to a lack of respectful or compassionate care [6, 7]. In some instances where women have opted for facility-based births, they have still reported dissatisfaction with their birth outcomes due to the way they were mis(treated) at the facilities [8]. This is indicative that respectful care is essential not only to promote uptake of facility-based deliveries but also to improve clinical birth outcomes and reduce complications.

The WHO Human Reproductive Programme (WHO-HRP) has prescribed recommendations on improving maternal health service delivery with respectful maternity care as an essential component of quality care [9]. The WHO defines Respectful Maternity Care (RMC) as the organisation and management of health systems in a manner that ensures respect for women’s sexual and reproductive health and human rights [10]. RMC, sometimes referred to as compassionate care, refers to care that emphasises the positive interpersonal interactions of parturients with health care providers and staff in a manner that maintains their dignity, privacy and confidentiality; ensures freedom from harm and mistreatment and enables informed choice and continuous support during labour and childbirth. The concept recognises that all women have the fundamental right to dignified and respectful care during childbirth and that mistreatment during childbirth is not only a blatant violation of women’s reproductive rights but is also a stringent disincentive for facility-based care and skilled birth attendance even in the absence of several other barriers of access. Whilst this is a fundamental right of all women, available evidence seems to suggest that certain vulnerable sub-groups of women, specifically younger, poorer, less educated, physically challenged, HIV-positive and ethnic minority women often face a health inequity in the enjoyment of RMC [11, 12]. For adolescents, this confers a higher chance of being denied RMC by reason of their characteristic young age, poorer and less educated status [13]. Additionally, provider moral biases against adolescents for their indulgence in early/pre-marital sex may also cause them to be treated with disrespect [8, 13]. This is a worrying event as adolescent parturients are proven to bear an excessively higher risk of negative pregnancy health outcomes than their older counterparts. Ideally, they should be treated with the utmost care and professionalism [14, 15].

Some interventions have sought to improve RMC in facilities using a variety of methods such as educating parturients on their SRH rights and seeking legal redress in some reported cases [16, 17]. However, these interventions do not often address the peculiar needs and challenges of adolescents. For example, adolescents may not necessarily be able to assert their rights due to their vulnerability and may not have the financial access to legal redress. There is therefore a need to review literature on interventions that are designed with specific attention towards providing RMC for adolescents and their successes and challenges. This is to help inform the design of future interventions in the delivery of quality maternity care for adolescents.

### Review aim

The overall aim of this systematic review is to synthesise the available evidence on respectful maternity care interventions targeted at reducing intrapartum mistreatment of adolescents.

### Specific objectives

The specific objectives of this review are as follows:

1. To review evidence on the types and characteristics of RMC interventions that have been specifically targeted at adolescents
2. To review evidence on the strategies, outcomes, gaps and challenges related to the implementation of RMC interventions for adolescents.

## Methods

This study protocol was registered with the International Prospective Register of Systematic Reviews (PROSPERO) with code CRD42020183440. This study protocol is being reported in accordance with the Preferred Reporting Items for Systematic Reviews and Meta-Analyses Protocols (PRISMA-P) statement [18] and checklist (Additional file 1).

### Eligibility criteria

Studies will be selected using the Population, Intervention, Comparator, Outcome and Study design (PICOS framework) [18].

### Participants

Only studies that are focused on adolescent parturients as a main study population or sub-analyses population of interest will be included. Adolescent parturient refers to anyone between 10 and 19 years old who has delivered within the past 6 months.

### Interventions

Any studies whose aim mentions interventions aimed towards providing respectful or compassionate care for adolescents will be included. Studies that focus on adolescent perspectives and experiences of quality of care will also be included.

### Comparators

Comparators will include studies that compare facilities or programmes that deliver the normal or standard quality of care for adolescents to facilities or programmes that are not specifically targeted at reducing intrapartum mistreatment.

### Outcome

The outcomes of interest are the reported experiences of RMC by participants. These outcomes include reported satisfaction with care and maternal and neonatal physical and psychosocial outcomes.

### Study design

Studies eligible for inclusion include relevant primary qualitative and quantitative research studies. These may include cross-sectional, cohort (prospective and retrospective), case control, experimental and intervention designs. Qualitative observations of respectful care experiences will also be included. Studies published in English and between January 1, 1990, and June 30, 2021, will be included. This timeline is selected to reflect the period from which the concept of respectful maternity care gained momentum in the 1990s to the most recent studies of 2021.

### Information sources and search strategy

The sources of information will be electronic databases including MEDLINE, PubMed, ScienceDirect, Cochrane, CINAHL, PsycINFO, Scopus, Google Scholar and Web of Science. Reference lists of selected studies will also be searched for relevant papers. Additionally, grey literature searches will be conducted on organisational websites such as World Health Organisation, White Ribbon Alliance, USAID and Population Council, a search strategy using medical subject headings (MeSH) on the terms ‘Intrapartum Mistreatment’, ‘Disrespect and Abuse’, ‘Respectful Maternity Care’, ‘Adolescents’, ‘Teenager’, ‘Pregnancy’ and ‘Compassionate care’ together with BOOLEAN operators (‘AND’/’OR’) will be used. The searches will be conducted by HH.

### Data extraction and management

All identified studies will be saved into the online-based Mendeley reference manager. This reference manager has been selected for this study as it allows orderly download and storage of the selected abstracts as well as any available full-text versions. It also allows shared access by all the reviewers. The relevant titles and abstracts will be independently screened by two reviewers HH and JM. Articles meeting the selection criteria will be retained for independent assessment against the selection criteria by HH and JM. A data extraction tool in Ms-Excel will be used to assess and extract the pertinent preliminary information from the available abstracts. Components of the tool will be used to extract the relevant data which include author(s) names, year of publication, study design and/or methodology, study population, intervention(s), study setting, geographic location and results. The final list of articles will be downloaded in full text for detailed review. A PRISMA flowchart will be used to demonstrate the process of screening and identification of articles to include in the systematic review, with reasons for exclusion noted. Any discrepancies that arise will be reassessed and resolved by the full team.

### Reporting quality in individual studies

Studies will be individually assessed for quality using the suitable Joanna Briggs Institute critical appraisal tool [19] for each study design. Criteria that will be assessed will include congruity between the study aims and objectives, its philosophical perspective and methodology as well as the analyses method used in the studies. Two authors (HH and JM) will review the studies against the eligibility criteria and the checklist independently. Discrepancies will be resolved by discussion, with the involvement of a third reviewer when there is a disagreement.

### Data analyses and synthesis

Preferred Reporting Items for Systematic Reviews and Meta-Analyses (PRISMA) guidelines 18 will be followed during the review. A combination of narrative and thematic synthesis is proposed as most suitable for achieving this review’s objectives which aim to describe the existing literature as well as identify the strategies, outcomes, gaps and challenges in previous interventions. The descriptive [20] narration will firstly summarise the methods, results and conclusions of the studies in prose. Subsequently, the running themes in the studies will be identified and grouped in a thematic analyses [21]. The most prominent and recurrent themes will then be identified and analysed. The characteristics and themes will also be summarised in a tabular form in addition to the prose narratives. If the included studies are sufficiently homogeneous (relating to study population, methodology, intervention and outcome), meta-analyses will be considered, using random-effects model in STATA version 16 software to account for between-study variability. If a meta-analysis is conducted, statistical heterogeneity will be assessed using the *X*^2^ test having a 10% significance level and quantified using the *I*^2^ statistic.

## Discussion

Some studies have demonstrated that RMC can be improved with beneficial outcomes to parturients, neonates and entire communities using a variety of interventions. A study in Kenya demonstrated an increase in respectful maternity care provision after implementing interventions that teach and encourage women to know and assert their sexual reproduction health rights [22, 23]. Some studies have also encouraged women to seek legal redress against their abusers [16]. Again, other studies have sought to educate communities on how to be custodians and support women against mistreatment and demand respectful care as a health right [24].

Despite this available evidence on the success of RMC interventions in reducing mistreatment and improving the quality of maternal care, little is known about interventions that work, or do not work especially for adolescents and vulnerable sub-groups of women who may not necessarily be able to benefit from these existing interventions. Adolescents for instance may be aware of their own SRH rights but may not be able to assert them due to their generally younger age. Additionally, they may not be able to seek legal redress due to financial constraints and may also not have support from the wider community due to widespread moral judgement against their engagement in early sex [25]. This gap exists and evidently creates a health inequity for adolescent parturients.

In order to overcome this gap, there is a need to investigate any available evidence on strategies that work best in promoting RMC for adolescents and other vulnerable sub-groups of women. In a group of women such as adolescents who bear an elevated risk to maternal mortality and morbidity [26, 27], it is essential that the highest level of quality peripartum care be provided to encourage facility-based, and also, improve clinical and psychosocial maternal and neonatal outcomes.

This review therefore contributes to efforts in the reduction of maternal mortality and morbidity especially amongst adolescents who are a key risk group. The review will provide a much-needed insight of what interventions have been put into place for adolescents, the challenges in their implementation, as well as the strategies that have led to their success. It will additionally help to identify the existing research and programmatic gaps as well as recommendations for any future research, interventions, policy and programmes.

### Dissemination

The results of this review will be submitted for open access publication. The results will also be submitted as part of a doctoral thesis and presented at conferences.

### Strengths and limitations

To the best of our knowledge, this review will be the first to synthesise evidence on RMC for adolescents. Additionally, it will include studies from a wide variety of relevant sources including grey literature sources.

## Supplementary Information

**Additional file 1.** PRISMA Checklist.

**Additional file 2.** Search Terms.

## Data Availability

Not applicable

## Abbreviations

ASRHR: Adolescent sexual reproductive health and rights
DHIMS: District Health Information Management Systems
HRP: Human reproduction programme
RMC: Respectful Maternity Care
SBA: Skilled birth attendance
SRHR: Sexual reproductive health and rights
WHO: World Health Organisation
