# Supplementary material for: Evidence on respectful maternity care for adolescents: a systematic review protocol
Source: Syst Rev. 2021 Oct 15;10:269. doi: 10.1186/s13643-021-01829-9 (PMC8520233; doi:10.1186/s13643-021-01829-9)
Supplement: Supplementary file 2 — Additional file 2. Search Terms. [file 13643_2021_1829_MOESM2_ESM.docx]

Additional File 3: Search Strategy for databases

Search terms:

1. Intrapartum Mistreatment
2. Disrespect and Abuse
3. Respectful Maternity Care
4. Compassionate Maternity Care
5. 1 OR 2 OR 3 OR 4
6. Adolescent
7. Teenager
8. “Intrapartum Mistreatment” and Adolescent (MeSH)
9. “Intrapartum Mistreatment” and Teenager (MeSH)
10. “Disrespect and Abuse” and Adolescent (MeSH)
11. “Disrespect and Abuse” and Teenager (MeSH)
12. “Respectful Maternity Care” and Adolescent (MeSH)
13. “Respectful Maternity Care” and Teenager (MeSH)
14. “Compassionate Maternity Care” and Adolescent (MeSH)
15. “Compassionate Maternity Care” and Teenager (MeSH)
16. 8 OR 9
17. 10 OR 11
18. 12 OR 13
19. 14 OR 15
20. 8 OR 9 OR 10 OR 11 OR 12 OR 13 OR 14 OR 15
21. Adolescent pregnancy
22. Teenage pregnancy
23. Adolescent pregnancy abuse
24. Adolescent pregnancy mistreatment
25. Teenage pregnancy mistreatment
26. 24 OR 26
27. 25 or 26
28. Young mothers
29. Young mothers mistreatment
30. Young mothers abuse health facilities
31. Adolescent abuse health facilities
32. 21 OR 34
33. 22 OR 34
